# Hyperactivation of Alk induces neonatal lethality in knock-in Alk^F1178L^ mice

**DOI:** 10.18632/oncotarget.1882

**Published:** 2014-04-02

**Authors:** Lucille Lopez-Delisle, Cécile Pierre-Eugène, Evelyne Bloch-Gallego, Marie-Christine Birling, Jean-Loup Duband, Estelle Durand, Thomas Bourgeois, Boris Matrot, Tania Sorg, Michel Huerre, Hamid Meziane, Michel J. Roux, Marie-France Champy, Jorge Gallego, Olivier Delattre, Isabelle Janoueix-Lerosey

**Affiliations:** ^1^ Inserm U830, Paris, France; ^2^ Institut Curie, Centre de Recherche, Paris, France; ^3^ Institut Cochin, Université Paris Descartes, CNRS (UMR 8104), Paris, France; ^4^ Inserm, U1016, Paris, France; ^5^ Institut Clinique de la Souris, Illkirch-Graffenstaden, France; ^6^ Sorbonne Universités, Université Pierre et Marie Curie-Paris 6, Paris, France; ^7^ CNRS, Laboratoire de Biologie du Développement, Paris, France; ^8^ PhenoPups SAS, Evry, France; ^9^ Inserm U1141, Hopital Robert Debré, Paris, France; ^10^ Institut Curie, Département de Pathologie, Paris, France

**Keywords:** ALK, brainstem, neonatal lethality, plethysmography, feeding difficulties

## Abstract

The *ALK* (Anaplastic Lymphoma Kinase) gene encodes a tyrosine kinase receptor preferentially expressed in the central and peripheral nervous systems. A syndromic presentation associating congenital neuroblastoma with severe encephalopathy and an abnormal shape of the brainstem has been described in patients harbouring *de novo* germline F1174V and F1245V *ALK* mutations. Here, we investigated the phenotype of knock-in (KI) mice bearing the Alk^F1178L^ mutation (F1174L in human). Although heterozygous KI mice did not reproduce the severe breathing and feeding difficulties observed in human patients, behavioral tests documented a reduced activity during dark phases and an increased anxiety of mutated mice. Matings of heterozygotes yielded the expected proportions of wild-type, heterozygotes and homozygotes at birth but a high neonatal lethality was noticed for homozygotes. We documented *Alk* expression in several motor nuclei of the brainstem involved in the control of sucking and swallowing. Evaluation of basic physiological functions 12 hours after birth revealed slightly more apneas but a dramatic reduced milk intake for homozygotes compared to control littermates. Overall, our data demonstrate that Alk activation above a critical threshold is not compatible with survival in mice, in agreement with the extremely severe phenotype of patients carrying aggressive *de novo ALK* germline mutations.

## INTRODUCTION

The *ALK* (Anaplastic Lymphoma Kinase) gene encodes a tyrosine kinase receptor that belongs to the insulin-receptor superfamily [1,2]. It is involved in many human cancers through a variety of mechanisms, including translocations, amplifications and activating point mutations [3,4]. In neuroblastoma, a pediatric cancer of the peripheral sympathetic nervous system (SNS), such mutations have been identified in both familial and sporadic cases with different spectra: whereas three hotspots of mutations have been described in sporadic cases, at positions F1245, R1275 and F1174, no germline mutation affecting the F1245 and F1174 residues has been reported in neuroblastoma families [5,6]. The R1275Q is the most frequent germline mutation observed in these forms [7,8]. The observation of missing germline mutations relative to the somatic repertoire suggested that a highest activity of these mutated forms may induce severe effects during embryonic development resulting in embryonal lethality. Yet, a syndromic presentation associating congenital neuroblastoma with severe encephalopathy and abnormal shape of the brainstem has now been described in two sporadic cases harbouring *de novo* germline F1174V and F1245V *ALK* mutations [9]. These patients presented with major feeding difficulties, associated with poor sucking and swallowing. They exhibited breathing difficulties with severe apneas and respiratory support was provided in both cases. They were also hypotonic and died before one year of age.

The involvement of the *ALK* gene in neuroblastoma and neurological disorders is indeed consistent with its preferential expression in the central and peripheral nervous systems that has been reported in mammals [10–12]. However, the precise function of full length ALK remains poorly understood. The *in vivo* function of ALK has been largely evaluated in the model organism *Drosophila melanogaster*. *Drosophila* embryos lacking dALK die due to abnormalities in the formation of gut musculature [13] and the dALK receptor is also involved in the neuronal circuit assembly of the visual system [14]. More recently, it has been shown that dALK plays critical roles in body size determination and associative olfactory learning [15] and that it permits central nervous system (CNS) growth under nutrient restriction [16]. In contrast to *Drosophila*, mice deficient in Alk are viable, fertile and display a normal appearance [17,18]. However, some abnormalities in behavioral tests have been reported. Analysis of another mouse strain inactivated for Alk revealed that mutant mice consumed more ethanol than wild-type (WT) mice and showed resistance to the ataxic effect of ethanol [19]. We recently described a prolonged neurogenesis in the SNS of knock-in (KI) mice bearing Alk R1279Q and F1178L activating mutations as well as a strong cooperation between Alk mutations and MYCN overexpression in neuroblastoma formation [34]. The studied mutations correspond to the ALK R1275Q and F1174L mutations observed in neuroblastoma patients, respectively.

In the present paper, we sought to determine whether KI Alk^F1178L^ mice phenocopy the severe neurological disorders observed in patients with *de novo* germline activating *ALK* mutations [9]. We analyzed in-depth basic physiological functions of heterozygous and/or homozygous KI Alk^F1178L^ animals, including behavior, breathing and feeding. We also investigated *Alk* expression in the brainstem, which is crucial for many basic functions and performed a complete histology analysis of homozygotes at birth. Altogether, our data strongly suggest that Alk activation above a critical threshold impairs survival in mice.

## RESULTS

### Heterozygous KI Alk^F1178L^ mice are viable and display few behavioral abnormalities

Heterozygous (HT) KI Alk^F1178L^ HT mice were generated at Institut Clinique de la Souris (Illkirch-Graffenstaden, France). Two animals were used to expand the line and consecutive backcrosses with C57Bl6/N WT animals were performed for at least 6 generations. *Alk* cDNA obtained from brain of HT mice showed equivalent expression of both alleles (data not shown). These HT animals had a normal physical appearence and were indistinguishable from WT littermates. Taking into account all backcrosses we obtained at weaning 46 % of HT (429 HT/928 animals). This is slightly below the expected ratio of 50% (chi-square test, p = 0.02).

**Figure 1:**
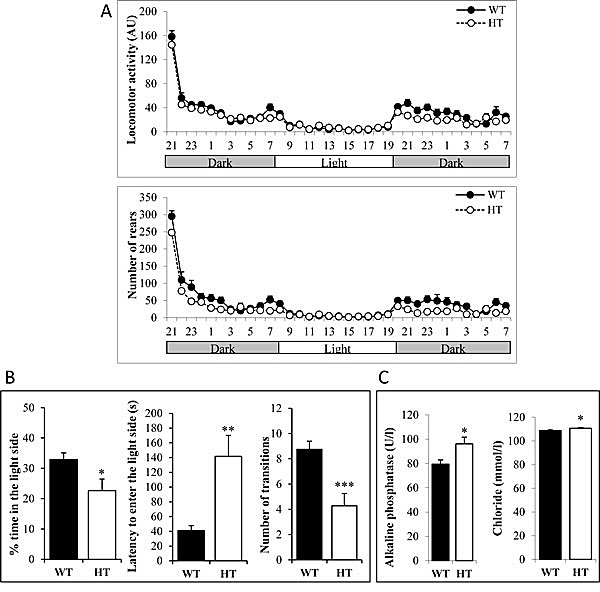
Behavioral abnormalities of heterozygous adult KI Alk^F1178L^ males A. Monitoring of locomotor activity and rears during dark and light phases. Each dot represents the mean of animals on one hour periods. The difference between genotypes was significant during the first dark phase for locomotor activity (p<0.05) and the number of rears was also significantly decreased in HT males during the dark phases (p<0.05). B. Results of the light/dark test indicate an increased anxiety of HT mice. C. Concentration of alkaline phosphatase and chloride in HT and WT mice. Error bars correspond to SEM, p-value for t-test *: <0.05, **: <0.01, ***: <0.001.

Abnormalities in behavioral tests have been noticed in Alk deficient mice [17]. We therefore submitted HT KI Alk^F1178L^ and WT male mice to a battery of tests including: (1) circadian activity and ingestive behaviors as well as modified SHIRPA for general health and gross neurological examination; (2) rotarod and grip tests for motor abilities and muscle strength; (3) slit lamp and topical endoscopy fundus imaging for analysis of the visual system; (4) hot plate test for pain sensitivity; (5) light/dark test for anxiety-related behavior; (6) tail suspension test for depression-like behavior; (7) auditory startle reflex reactivity and pre-pulse inhibition test for sensorimotor gating; (8) Y-maze test and pavlovian fear conditioning tests for learning and memory and (9) blood analysis. HT mice had good general health and did not show any obvious sign of altered sensory or vestibular function. However, we could detect significant differences compared to WT in a subset of tests. For the circadian activity, HT were less active during the dark phases for both locomotor activity and rears (Figure 1A). HT mice showed significantly increased muscle strength when animals were allowed to grip with all paws (WT: 7.24 ± 0.17 g per gram; HT: 7.93 ± 0.23 g per gram; p<0.05). Minor abnormalities of the visual system, *i. e.* presence of a small zone of higher reflectance under one of the main blood vessels and/or a small white spot under a vein, were observed in 7 out of the 11 HT mice. During the dark/light test, HT spent less time in the light side, waited longer before going for the first time in the light side and did less light/dark transitions (Figure 1B). Blood analysis showed that HT mice displayed slightly higher alkaline phosphatase and chloride concentrations compared to WT littermates (Figure 1C). HT did not show any difference with WT in the other tests, in particular in the Y-maze and tail suspension tests (Supplementary figure 1A-1B, respectively). Altogether, these observations indicate that mice bearing the Alk^F1178L^ activating mutation at the heterozygous status displayed an increased anxiety in the dark/light test but no severe neurological alterations.

### High neonatal lethality of homozygous KI Alk^F1178L^ mice

**Figure 2:**
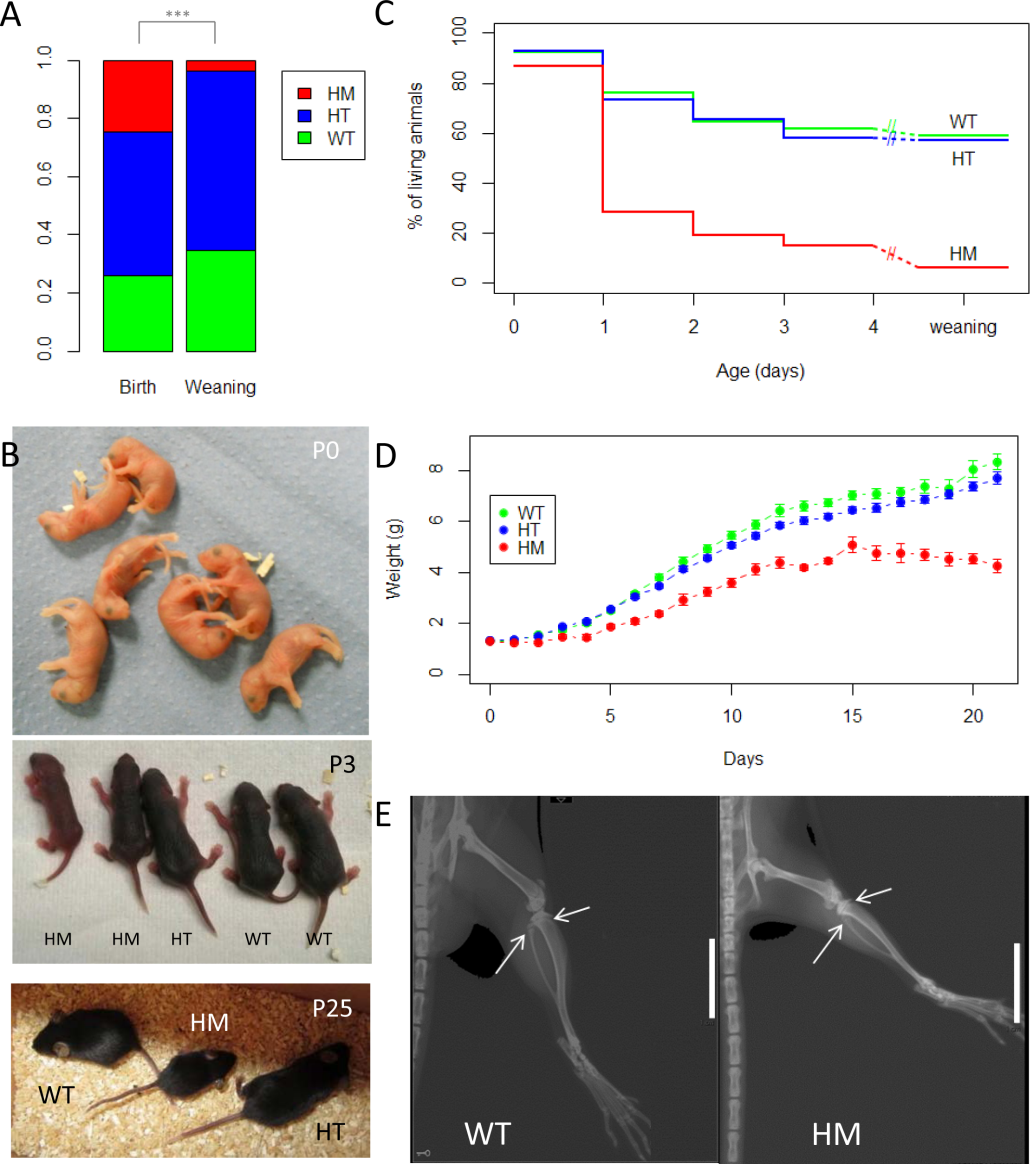
High neonatal lethality of homozygous KI Alk^F1178L^ mice and growth delay of surviving homozygotes A. Breedings between HT were performed to obtain HM. Results of genotyping at birth (n=502) and at weaning (n=158) are shown. At birth: WT: n=131, HT: n=248 and HM: n=123. At weaning: WT: n=55, HT: n=97 and HM: n=6. p-value for the chi-square test: 7.10^−8^ B. Representative pictures of litters at different ages. At birth, the 3 genotypes are indistinguishable (WT, n=2; HT, n=2; HM, n=3). At P3, the subset of HM that survive may be identified due to their reduced size and delay in skin pigmentation. At P25, the surviving HM still present with a reduced size. C. Survival curves indicating the time of death for HM. D. Mean weight of each genotype from birth to weaning. Error bars correspond to SEM. E. X-ray of back paw of one WT and one HM young adults. Epiphyseal plates are indicated by arrows and present in both animals. White bar: 1 cm.

We hypothetized that mice may be more resistant to ALK activation than humans. With the aim to potentially increase the phenotype induced by activated Alk, matings between HT were performed to obtain homozygous (HM) KI Alk^F1178L^ mice. At birth, a quarter of all genotyped animals were HM (Figure 2A) and these animals could not be distinguished from WT and HT (Figure 2B, upper panel). This demonstrates that this mutation was not embryonic lethal in mice even when homozygous. However, at weaning HM animals represented only 3.8 % of all animals (Figure 2A). Therefore a homozygous Alk^F1178L^ mutation induces a strong neonatal lethality. Analysis of the precise day of death showed a major peak of lethality between 24 hours (P1) and 48 hours (P2) after birth (Figure 2C). In addition, we noticed that the subset of HM that survived more than two days were smaller, lighter than WT and presented with a delay in skin pigmentation and fur growth (Figure 2B, middle and lower panels and figure 2D). In order to investigate whether this growth delay was associated with a delay in bone maturation, we performed whole-body X-rays of one HM and one WT at 6 weeks of age. The HM showed shorter femoral and tibial diaphyses, in accordance with the smaller size of the HM population described above (Figure 2E). Still, observation of ossification of epiphyseal cartilages at inferior extremity of femur and superior extremity of tibia showed no maturation delay in the HM.

### At birth, homozygous KI Alk^F1178L^ mice do not present abnormalities in brainstem and whole-body histology

**Figure 3:**
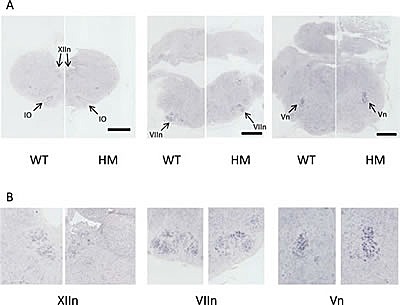
Absence of abnormalities of the brainstem nuclei expressing *Alk* in HM KI Alk^F1178L^ mice A. *In situ* hybridization on coronal sections (rostral to caudal) of brainstem of WT and HM embryos at E18.5 with an *Alk* probe. Black scale bar: 0.5 mm. B. Magnification images of the cranial nerve motor nuclei. IO: Inferior Olivary nucleus; XIIn: hypoglossal motor nucleus; VIIn: facial motor nucleus; Vn: trigeminal motor nucleus.

The syndromic patients with *de novo* germline activating *ALK* mutations presented with an enlarged medulla obloganta evidenced by magnetic resonance imaging [9]. We therefore sought to document *Alk* expression in the brainstem and determine whether KI Alk^F1178L^ mice exhibited abnormalities of this structure. *In situ* hybridization (ISH) of *Alk* was performed on fixed brainstems of WT mice at different stages from E15.5 to P0. *Alk* expression was consistently detected in the inferior olivary nucleus as well as in different cranial nerve motor nuclei including the hypoglossal (XIIn), ambiguus, facial (VIIn) and trigeminal (Vn) nuclei (Figure 3 and data not shown). These nuclei are involved in the control of sucking and swallowing. The same pattern of expression was observed in the HM embryos at E18.5 (Figure 3). We confirmed by Western blot that the mutated Alk protein was indeed expressed in the brainstem of HM mice at similar level compared to WT littermates (Supplementary figure 2). Our ISH experiments also revealed that the motor nuclei expressing *Alk* were present in HM mice with non altered shape and size compared with control littermates.

In order to find what could be responsible for HM death, we performed a whole-body histology analysis with HES (Hematoxylin-Eosin-Safran) staining on 5 HM and 5 WT at birth (Supplementary figure 3A). This analysis showed that all organs were normal in terms of size, shape and cellular aspect. A specific examination of the adrenal gland did not reveal any abnormality in the HM mice.

### Minor breathing abnormalities but dramatic decreased milk intake of homozygous KI Alk^F1178L^ mice at birth

**Figure 4:**
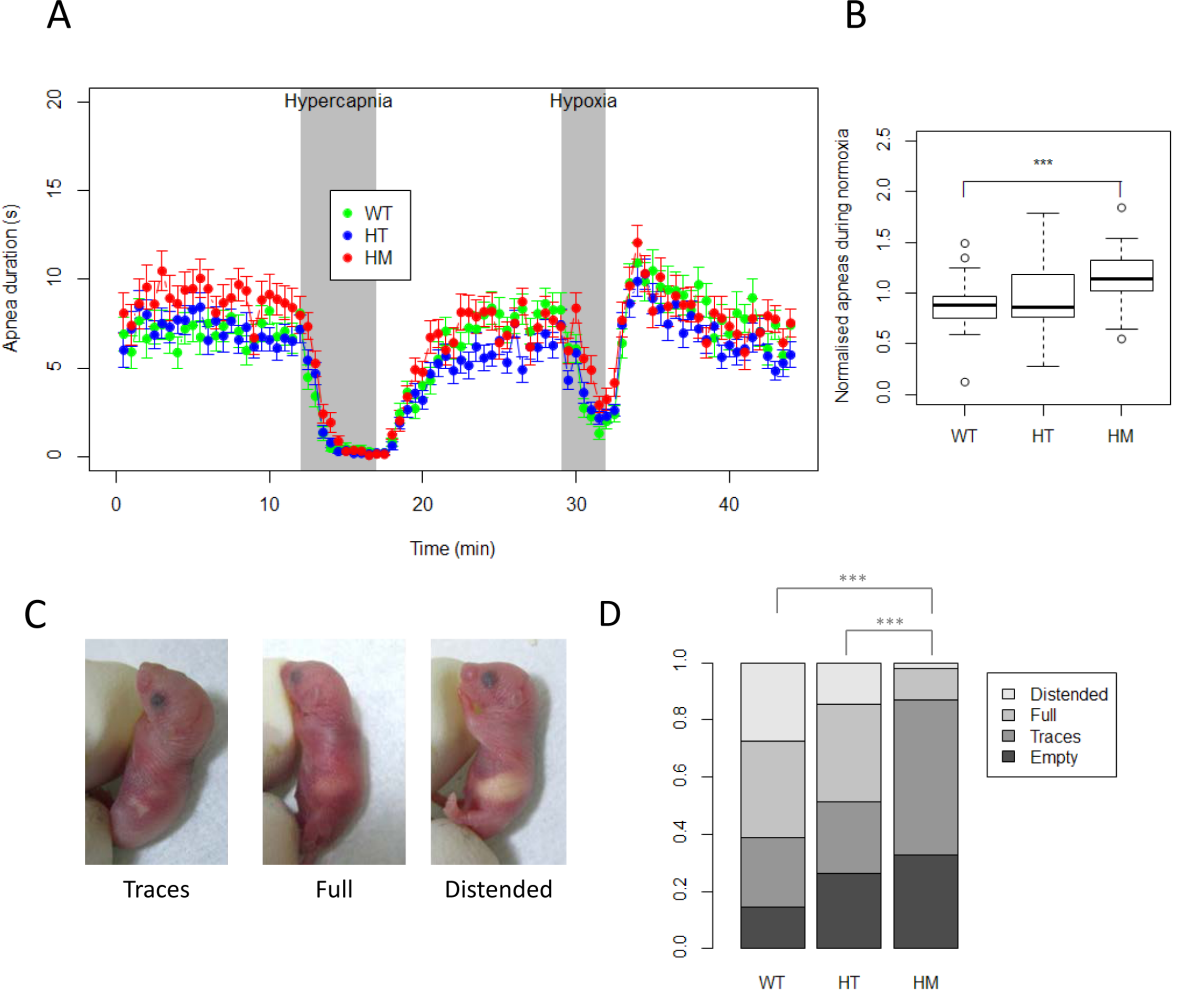
Analysis of basic physiological functions at birth reveals a strong decreased milk intake for homozygous KI Alk^F1178L^ mice A. Apneas were recorded by whole-body plethysmography on 94 newborn mice (WT, n=27; HT, n=41; HM, n=26) under normoxia, hypercapnia and hypoxia. Means of apnea duration for all genotypes are shown in the different conditions. Each dot represents a period of 30 seconds. Error bars correspond to SEM. B. Normalized apneas duration during normoxia are shown. Each apneas duration for the 10 min of normoxia was normalized by the mean of the duration in the litter. p-value for t-test between WT and HM = 0.00055. C. Representative pictures of milk intake in the stomach of newborn mice scored as traces (left panel), full (middle panel) or distended (right panel). D. Repartition of different milk intake scores for each genotype (WT, n= 62; HT, n=103; HM, n=46), p-values for the chi-square tests ***: <0.001.

Major breathing and feeding difficulties characterized the children with *de novo* germline activating *ALK* mutations and these patients required respiratory support and tube feeding [9]. We were therefore interested in the capacity of HM KI Alk^F1178L^ mice to breath and feed normally. First, we investigated breathing of the HM mice to determine whether they showed defects, such as many apneas similarly to the patients. We analyzed 94 pups around 12 hours after birth using whole-body plethysmography, in three different conditions including normoxia, hypercapnia and hypoxia. No differences were detected in the plethysmography measurements, *i.e.* tidal volume (V_T_), breath duration (T_TOT_)_,_ and minute ventilation (V_E_, calculated as V_T_/T_TOT_) (Supplementary figure 4A-4C) showing that HM breathed correctly with the same rate and volume compared to controls. We documented that HM responded correctly to hypercapnia and hypoxia (Figure 4A and Supplementary figure 4A-4C). However, we noticed that HM spent about 30% more time in apneas in normoxia condition (Figure 4B). Movements were also recorded during the three conditions. In particular, activity duration is indicative of the arousal and defensive response to hypercapnia. We observed that this movement response to hypercapnia was markedly disrupted in HM and HT mice compared to WT mice (Supplementary figure 4D). In contrast, no difference was recorded between the three genotypes neither in hypoxia nor in normoxia.

The measurement of body weight on these 94 animals (WT, HT, HM) showed that there was no difference according to the genotype at birth. It was confirmed on a larger series of 236 pups (Figure 2D). The interscapular skin temperature of the 94 pups was recorded when the mother just went outside the nest. This measurement did not show any difference that could have suggested that HM mice were excluded from nest (data not shown). On the same cohort of 94 pups, the heart rate was similar for the three genotypes (data not shown).

To investigate the feeding capacity of the newborn mice, the stomach of animals born from HT intercrosses was scored at birth into 4 levels: empty, traces, full or distended (Figure 4C). This analysis showed that whereas about a quarter of WT presented with a distended stomach only one HM out of 46 was classified in this category (Figure 4D). Moreover the proportion of animals having at least a full stomach was 61% for WT, 48% for HT but only 13% for HM. In conclusion, this analysis demonstrates that HM did slightly more apneas than littermate controls but this difference may hardly account for the neonatal lethality. Nevertheless, the HM exhibited a dramatic reduced milk intake consistent with the phenotype observed in patients.

## DISCUSSION

In the present paper, we performed an in-depth phenotyping analysis of KI Alk^F1178L^ mice. In particular, we sought to determine whether these mice reproduce the severe neurological disorders observed in patients with *de novo* heterozygous germline activating *ALK* mutations. Our data clearly indicate that mice exhibiting one mutated Alk F1178L allele did not display severe abnormalities related to those observed in the patient bearing an ALK F1174V mutation. This difference is unlikely due to the difference in the amino acid variation. The F1178 residue in the murine receptor corresponds to the F1174 position in the human receptor and the kinase domains of both receptors exhibit only four differences. The F1174 residue lies in the alpha C helix [3] and it has been suggested that a F to L change will result in an increased affinity for ATP [20]. The F1174L mutation has been shown to be a ligand-independent activating mutation [21]. The L and V residues present with the same physicochemical properties and are expected to induce the same consequences on the receptor's activity.

We recorded few abnormalities of heterozygous KI Alk^F1178L^ mice in behavioral tests, including a reduced activity during dark phases and an increased anxiety in the dark/light test compared to control littermates. However, we did not notice any abnormalities either in the learning and memory function, or in the susceptibility to despair. A few anomalies have also been reported in homozygous KO Alk mice [17] and double homozygous KO Alk/Ltk mice [18]. In the first study, authors reported an antidepressant profile, *i.e.* a decreased susceptibility to despair of KO Alk mice. No effect of Alk inhibition was detected on the locomotor activity and the anxiety. Results of the second study, suggested a decreased anxiety for Alk mutants and an increased performance in spatial memory. Interestingly, this phenotype is consistent with the one described in *Drosophila*, since dAlk inhibition was shown to enhance olfactory associative learning in this model organism [15]. It therefore appears that whereas Alk inhibition decreases anxiety in mice, Alk activation induces an opposite effect. We could not demonstrate effects of Alk activation on cognitive functions in our model. However, behavioral tests were performed only on HT KI Alk^F1178L^ mice and not on HM mice, due to the small number of surviving HM animals.

Although the HT mice did not exhibit strong phenotypes, we documented a high neonatal lethality of HM KI Alk^F1178L^ mice. We excluded an embryonic lethality since the expected proportions of the various genotypes were observed at birth from heterozygotes intercrosses. The newborn mutants could not be distinguished from the control littermates, indicating that Alk activation did not induce effects during *in utero* development that would be macroscopically visible at birth. We also obtained and characterized another KI Alk mouse line bearing a R1279Q mutation (corresponding to the R1275Q mutation in neuroblastoma patients). We did not observe any difference in the apparent phenotype and survival of R1279Q HT and HM animals compared to WT littermates. Breedings of HT provided the expected proportions of the different genotypes at weaning (n=26 WT, n=57 HT and n=26 HM, corresponding to 24%, 52% and 24% of all animals, respectively). This demonstrates that, in contrast to the F1178L mutation, the R1279Q mutation did not induce a neonatal lethality in mice. These differences between both *Alk* mutations highly suggest a threshold of Alk activation compatible with survival. We recently bred KI Alk^F1178L^ mice with mice expressing a truncated Alk receptor without a functional kinase domain. We therefore got hemizygote animals in which only one mutated Alk F1178L allele is expressed, in the absence of WT Alk. These animals did not show post-natal lethality (data not shown). This indicates that the absolute level of activated Alk, rather than a balanced expression between the mutant and WT forms is critical for survival. Furthermore the highest impact of the F1178L mutation on mice survival is consistent with the higher oncogenic potential of the F1178L mutation compared to the R1279Q mutation that we recently demonstrated *in vivo* [34]. Of interest, mutation at position 1245 in the human syndrome associating neuroblastoma and severe encephalopathy corresponds to the third hotspot of mutation reported exclusively at the somatic level in sporadic neuroblastoma and not described at the germline level in neuroblastoma families. One can predict that such a mutation would also impair survival in mice. Finally, neither developmental abnormalities nor neurological disorders have been reported in patients with *ALK* germline activating mutations observed in a context of familial neuroblastoma, suggesting that these mutations are indeed less aggressive.

Since the children presenting with *de novo* germline F1174V and F1245V activating ALK mutations displayed abnormal shape of the brainstem, we investigated this structure in KI Alk^F1178L^ mice. We documented high expression of *Alk* mRNA in brainstem motor nuclei involved in control of sucking and swallowing. However no differences in size and shape of these nuclei could be evidenced between mutant and WT mice. We performed as well a global histology analysis of newborn mice that did not reveal any overt abnormalities. Only a focused analysis of the SNS ganglia indicated an enlargement of the superior cervical and stellate ganglia in Alk R1279Q and F1178L knock-in mice [34]. Therefore it appears that Alk activation does not induce morphological abnormalities of the brainstem or vital organs that could account for the neonatal lethality of HM mice exhibiting the F1178 mutation.

With respect to the breathing function, KI Alk^F1178L^ mutant newborns were never seen cyanosed and only exhibited a modest increase in apneas duration compared to WT mice. These modest abnormalities are unlikely to explain the cause of HM animals death. This results strongly contrasts with the dramatic phenotype associated with determined mutations of Phox2b, which regulates Alk expression [22] and is also associated with neuroblastoma [5,23]. Specifically, Phox2b KI mice with polyalanine expansion died very soon after birth from major breathing difficulties and huge apneas [24].

Whereas breathing was not strongly affected in mutant newborns, we documented that feeding was severely impaired in KI Alk^F1178L^ HM. This observation may be closely related to the feeding difficulties that characterized the patients. In mice, it remains to be determined whether the reduced milk intake of mutant newborns results from specific sucking or swallowing defects or may be linked to a difficult access to the udder in the litter. It is of interest to mention that we never observed milk in the lungs of the HM newborns that were subjected to whole-body histology analysis, which suggests that these animals did not swallow the wrong way. Although we did not observe morphological anomalies of the brainstem nuclei expressing *Alk* and controlling sucking and swallowing we cannot exclude that Alk activation may functionally affect a subset of these neurons leading to a deficient milk intake. Another hypothesis would be that the feeding difficulties may not be related to the brainstem functions but to other CNS structures expressing the Alk receptor and playing a role in feeding and/or appetite regulation. Indeed, data of the literature and from our analysis indicate that Alk expression in mouse brain is not restricted to the brainstem [10-12]. In particular, it is expressed in the olfactory bulb. Impaired olfactory neurogenesis may induce olfactory deficits that could affect feeding of newborns. However, we did not observe any overt difference in HES of the olfactory bulb between WT and HM (Supplementary figure 3B), which is not in favour of such an hypothesis.

Overall, our data shed light on the activity-dependent and crucial role of Alk in the control of essential physiological functions in mice, in agreement with the extremely severe neurological disorders observed in patients carrying the most aggressive *ALK* germline mutations.

## METHODS

### Mouse line construction and genotyping

The construction of the mouse KI Alk^F1178L^ line as well as genotyping conditions are described elsewhere [34]. Briefly, the KI Alk^F1178L^ allele differs from the WT allele by an insertion of 75 pb in the 22^nd^ intron including a loxP site, a substitution TTC>CTC in the codon for the F1178 amino acid and another insertion of 75 pb in the 23^rd^ intron including a lox511 site. The genotyping primers Lf4735 (5' GTCGGCAGGAGATTTCAGAGACCA 3') and Mr4736 (5' GCAGGAGTTGAATTAGCGGGAAAAG 3') are flanking the first insertion. It allows to discriminate the WT allele (374 bp) and the KI Alk^F1178L^ allele (449 bp).

The care and use of animals further used in this study was strictly applying European and National Regulation in force for the Protection of Vertebrate Animals used for Experimental and other Scientific Purposes (Directive 86/609).

### Phenotypic analysis of adult KI Alk^F1178L^ males

This analysis was performed by Institut Clinique de la Souris (Illkirch-Graffenstaden, France). Twelve WT and 11 KI Alk^F1178L^ HT male mice were used for this study. They were allowed at least one week acclimation in phenotyping area with controlled temperature (21-22°C) under a 12-12 light-dark cycle, with food and water available ad libitum. Testing started at 11-12 week-old and was carried out in accordance with European institutional guidelines. Modified SHIRPA [25], rotarod, grip, hot plate and tail suspension tests, auditory startle reflex reactivity and prepulse inhibition test, Y-maze spontaneous alternation task [26,27] and Pavlovian fear conditioning [28] were performed as previously described. Briefly, for circadian activity and ingestive behaviors, spontaneous locomotor activity and rears were measured using 24 individual boxes equipped with infra-red captors. The quantity of water and food consumed was measured during the test period using automated pellet feeder and lickometer (Imetronic, Pessac, France). Mice were tested for 32 hours in order to measure habituation to the apparatus as well as nocturnal and diurnal activities. For visual clinical observations, the anterior segment (cornea, pupil, lens) of the eye was examined on vigil mice with a slit lamp (model 990 5X;CSO, Luneau, France) before and after pupil dilatation with a drop of 0.5% tropicamide (Novartis). The posterior segment (retina) was observed through topical endoscopy fundus imaging [29]. For blood analysis, blood was collected at 9.00 am on non fasted mice by retro orbital puncture under isoflurane anaesthesia. Blood chemistry was performed on an OLYMPUS AU-400 automated laboratory work station (Olympus France SA, Rungis, France) using commercial reagent (Olympus Diagnostica GmbH, Lismeehan, Ireland). A complete blood count was performed on an Advia 120 Vet (Siemens). Plasma immunoglobulins (IgG subclasses, IgA, IgM and IgE) were measured by immunoanalysis according to the Europhenome procedures (http://www.europhenome.org/). More detailed protocols are available on request. All data are expressed as mean ± SEM. Comparisons between HT and WT mice were performed using a Student t test. A statistically significant difference was considered for p<0.05.

### Whole-body mouse X-Ray analysis

High-resolution X-ray (100 microns pixel size) analysis was performed using a Senograph Essential apparatus (GE Healthcare).

### Histological analysis

For whole body histology, animals of 9-18 hours of life were killed with isoflurane, then fixed in a mix of ethanol-acetic acid-formol for 2 days, transfered in 70% ethanol and paraffin-embedded. Serial sagittal 10 μm sections were stained with HES. Pictures were taken using a Philips Image Management System 2.1 RA device.

For brainstem histology and *in situ* hybridization, embryos were harvested and brains were fixed by immersion in 4% paraformaldehyde overnight at 4°C. Brains were washed in PBS then in PBS-15% sucrose before inclusion in PBS-10% gelatin-15% sucrose and freezed at −20°C. Twenty μm slides were cut with a cryostat at −20°C then stored or stained. Images were taken using the Philips device. *In situ* hybridization was performed as previously described [30] with an *Alk* probe corresponding to positions 523 to 1356 of the *Alk* cDNA NM_007432.2. The *Alk* riboprobe was synthesized using the DIG RNA Labeling Kit (Roche) as specified by the manufacturer.

### Breathing analysis of newborn mice

Breathing variables were measured noninvasively using a battery of four whole-body flow barometric plethysmographs as previously described [31,33]. Plethysmograph chambers were immersed in a thermoregulated water-bath that maintained their temperature at 33°C, which corresponds to thermoneutrality in newborn rodents. The pups were taken randomly from the litter and underwent a hypercapnic and a hypoxic test separated by a normoxic period. After 12-min normoxia, hypercapnia was achieved by switching the airflow through the plethysmograph to 8% CO_2_ + 21% O_2_ + 71% N_2_ at the same flow rate (200 ml/min per chamber) for 5 min, after which the flow was switched back to normoxia for 12 min. Then hypoxia was achieved by switching the airflow through the plethysmograph to 10% O_2_ + 90% N_2_ at the same flow rate (200 ml/min per chamber) for 3 min, after which the flow was switched back to normoxia for another 12 min (total duration of the session: 44 min). Breath duration (T_TOT_, s), tidal volume (V_T_, μl/g), and minute ventilation (V_E_, calculated as V_T_/T_TOT_, μl/s/g) were calculated on apnea-free periods. The baseline (*i.e.* normoxia condition) levels for T_TOT,_ V_T_ and V_E_ were calculated as the mean value averaged on breath-by-breath values over the 10-min of air-breathing preceding the hypercapnic exposure. Apneas were defined as ventilatory pauses longer than 0.9 s, and determined using an automatic classification method adapted from Matrot *et al* [32]. Total apnea duration was calculated over successive 30-sec periods. Activity was detected based on large disturbances in the respiratory signal caused by the combined effects of positional changes inside the chamber and changes in breathing pattern [32]. Total movement duration was calculated over successive 30-sec periods.

### Statistical analysis

Statistical analyses were conducted using R (http://www.R-project.org).

## SUPPLEMENTARY FIGURES


