# Supplementary material for: Hyperactivation of Alk induces neonatal lethality in knock-in AlkF1178L mice
Source: Oncotarget. 2014 Apr 2;5(9):2703–13. doi: 10.18632/oncotarget.1882 (PMC4058038; doi:10.18632/oncotarget.1882)
Supplement: Supplementary file 1 [file oncotarget-05-2703-s001.pdf]

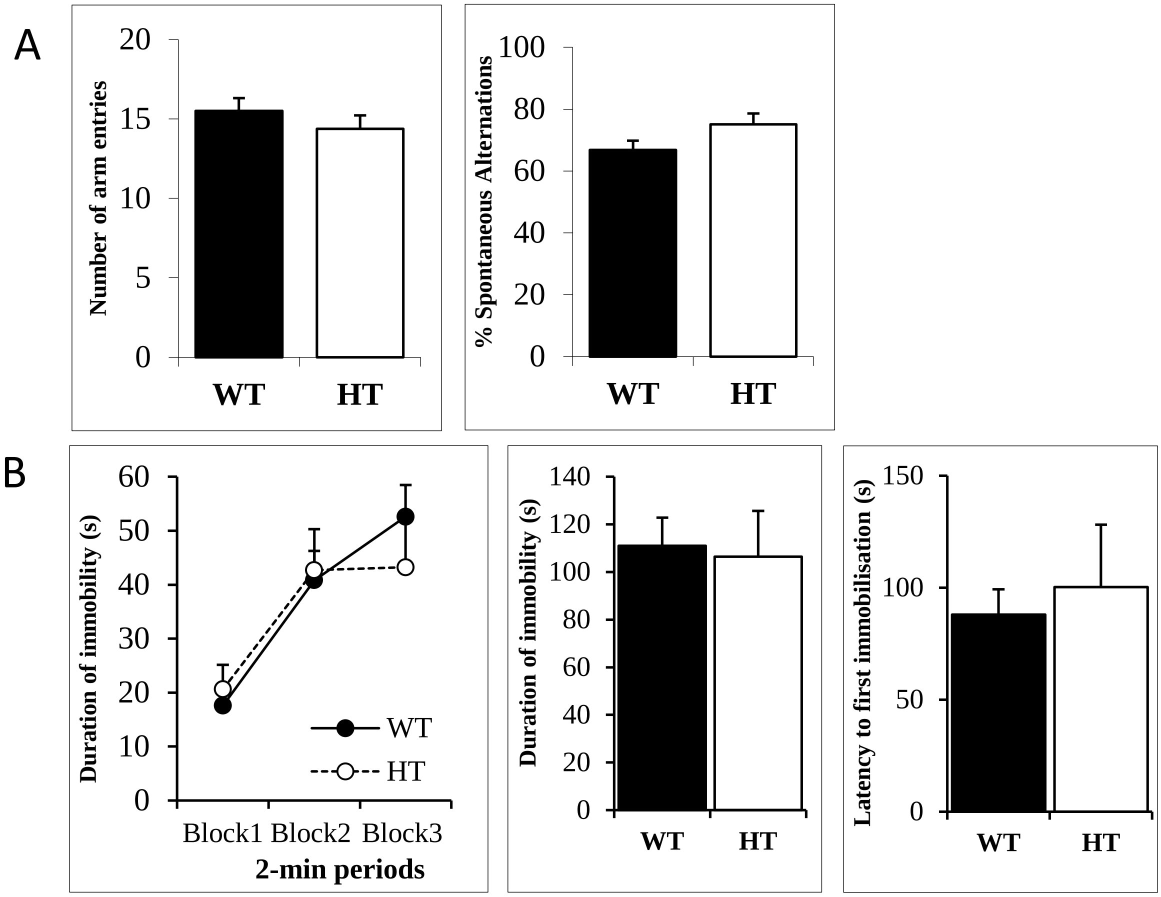

Supplementary figure 1: Results of Y-maze (A) and tail suspension (B) tests. Values are group means ± SEM.

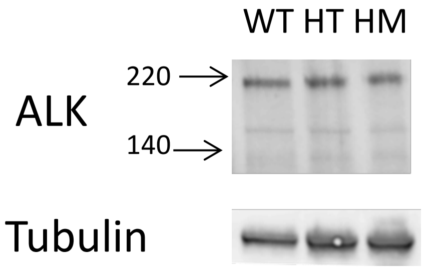

Supplementary figure 2: Alk expression in the brainstem of HT or HM KI Alk^{F1178L} mice compared to WT littermate. Alk expression was investigated by Western blot using the anti-RECA antibody (Bernard-Pierrot *et al*, J Biol Chem. 2002) on protein lysates from brainstem of adult littermates. Tubulin (HRP coupled antibody from Proteintech) was used as a standard.

A

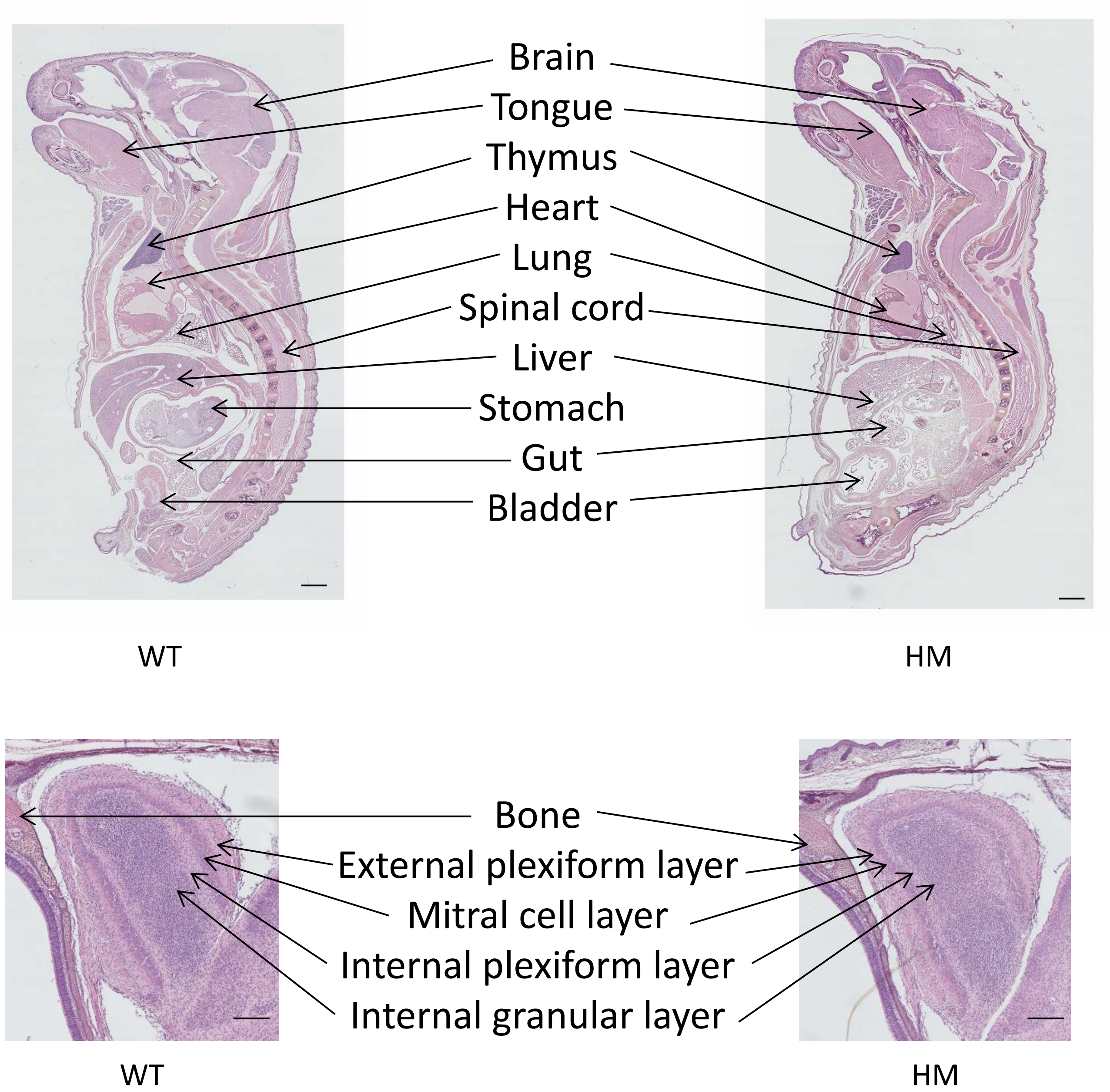

Supplementary figure 3: Histology of WT and HM KI Alk^{F1178L} mice at birth. A. Global histology. Median sagittal slides of WT and HM mice were stained with HES. Representative images are shown for one animal of each genotype. Scale bar: 1 mm. B. HES staining of the olfactory bulb of WT and HM mice. Scale bar: 0.2 mm.

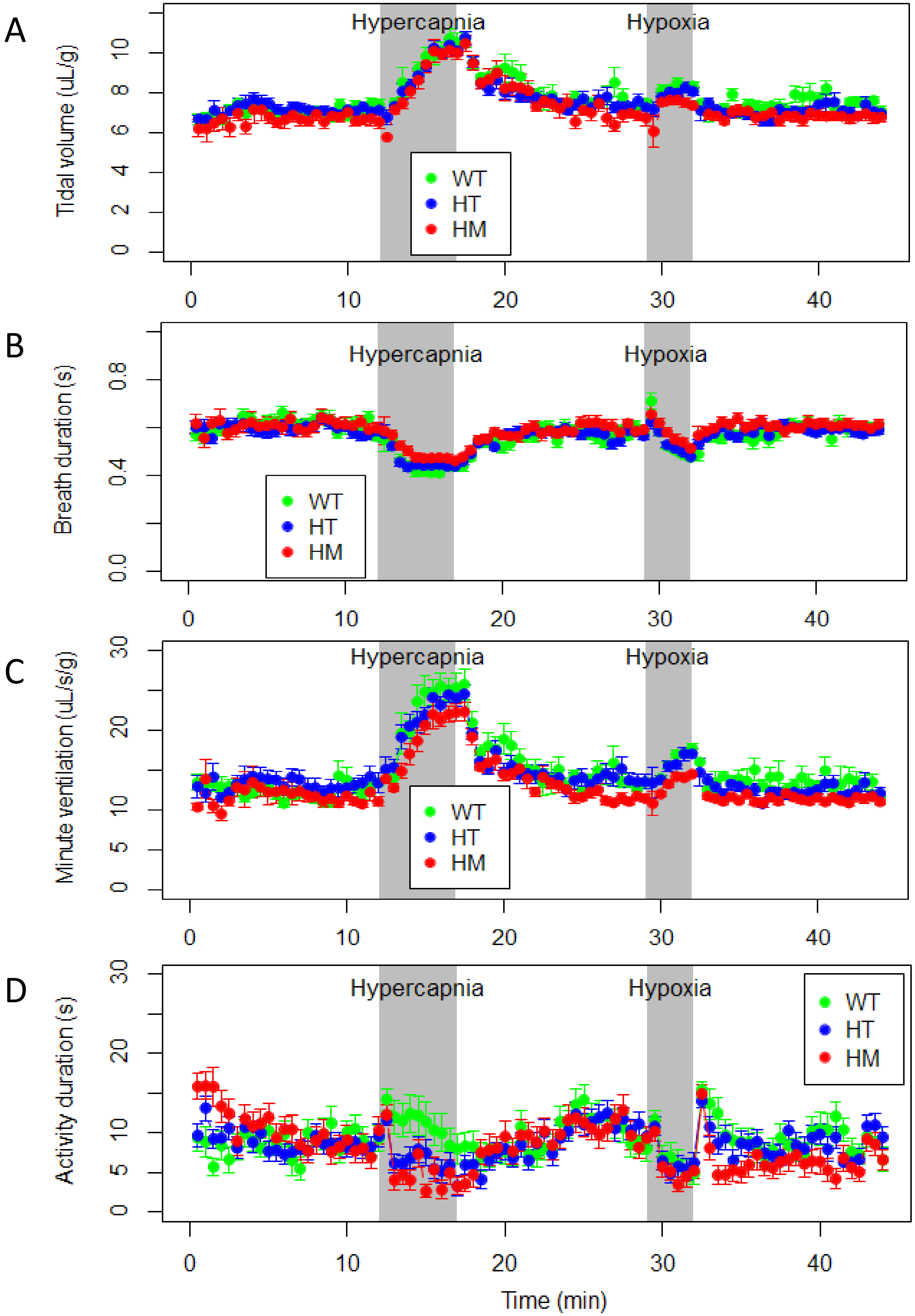

A
B
C
D
Hypercapnia
Hypoxia
WT
HT
HM
Tidal volume (uL/g)
Breath duration (s)
Minute ventilation (uL/s/g)
Activity duration (s)
Time (min)

Supplementary figure 4: Whole-body plethysmography analysis at birth. Tidal volume (V_T, A), breath duration (T_{TOT}, B), minute ventilation ($V_E = V_T/T_{TOT}$, C) and activity duration (D) were measured in HM (n=27), HT (n=41) and WT (white bar, n=26) pups. Values are group means for a period of 30 seconds and error bars represent SEM.
